# Supplementary material for: CD161, a promising prognostic biomarker in hepatocellular carcinoma, correlates with immune infiltration
Source: PeerJ. 2025 Mar 17;13:e19055. doi: 10.7717/peerj.19055 (PMC11925045; doi:10.7717/peerj.19055)
Supplement: Supplemental Information 1 [file peerj-13-19055-s001.docx]

Tabel S1. Summary of clinicopathologic data in TCGA and GSE14520.

| Variable | | TCGA set  (n=365) | GSE14520 set  (n=221) |
| --- | --- | --- | --- |
| Age(years) | ≤70 | 292 | 213 |
|  | >70 | 73 | 8 |
| Gender | Male | 246 | 191 |
|  | Female | 119 | 30 |
| TNM stage | Ⅰ+Ⅱ | 254 | 170 |
|  | Ⅲ+Ⅳ/Ⅲ | 87 | 49 |
|  | NA | 24 | 2 |
| Main tumor size (cm) | ≤5 | NA | 140 |
|  | >5 | NA | 80 |
|  | NA | NA | 1 |
| BCLC stage | 0/A | NA | 168 |
|  | B/C | NA | 51 |
|  | NA | NA | 2 |
| Survival status | Alive | 235 | 136 |
|  | Dead | 130 | 85 |
