# Supplementary material for: CD161, a promising prognostic biomarker in hepatocellular carcinoma, correlates with immune infiltration
Source: PeerJ. 2025 Mar 17;13:e19055. doi: 10.7717/peerj.19055 (PMC11925045; doi:10.7717/peerj.19055)
Supplement: Supplemental Information 2 [file peerj-13-19055-s002.docx]

Table S2. Therapeutic efficacy of the different group in GSE140901.

|  | High group (n=12) | Low group(n=12) | P value |
| --- | --- | --- | --- |
| PR | 4 | 2 |  |
| SD | 5 | 5 |  |
| PD | 3 | 5 |  |
| ORR | 33.3% | 16.7% | 0.640 |
| DCR | 75.0% | 58.3% | 0.667 |
