# Supplementary material for: CD161, a promising prognostic biomarker in hepatocellular carcinoma, correlates with immune infiltration
Source: PeerJ. 2025 Mar 17;13:e19055. doi: 10.7717/peerj.19055 (PMC11925045; doi:10.7717/peerj.19055)
Supplement: Supplemental Information 3 [file peerj-13-19055-s003.docx]

Table S3. The summary of CD161's role in different types of cancers and its potential molecular.

| Cancer types | Subject | Methods | Prognostic significance | Possible mechanisms | Reference |
| --- | --- | --- | --- | --- | --- |
| Breast cancer | Tissue/ cancer cells | mRNA | Positive | KLRB1/CD161 suppresses the proliferation, invasive and migrative abilities. KLRB1/CD161 enhance the anti-cancer immunity. | Huang G, et al ^[1]^ and He JR,et.^[2]^ |
| Oral squamous cell carcinoma | Tissue | IHC/ immunofluorescence | Positive | CD161/LLT1 influences the OSCC tumor immune microenvironment. | Hu X, et al. ^[3]^ |
| HPV-Driven Oropharyngeal Cancer | Tissue | Single-cell RNA-seq | Positive | KLRB1(CD161) boost the anti-tumor immune responses. | Wei Y, et al.^[4]^ and Cha J, et al^[5]^ |
| Pancreatic cancer | Tissue | Immunofluorescence  staining | Positive | Promote cytokine secretion, and enhance anti-tumor immunity | Chen Q,et al.^[6]^ |
| Lung cancer | Tissue/  cancer cells | mRNA | Positive | KLRB1 regulates lung adenocarcinoma cell proliferation and metastasis through the MAPK/ERK pathway. Down-regulation of KLRB1 is associated with increased cell growth, metastasis, poor prognosis, as well as a dysfunctional immune microenvironment. | Xu S, et al.^[7]^ and Chen JL,et al.^[8]^ |
| Gliomas | Tissue/  cancer cells | Single-cell RNA-seq/Co-Culture | Negative | KLRB1 inhibits the T cell- mediated immune response. | Mathewson ND, et al ^[9]^ and Wang Di, et al. ^[10]^ |
| Hematological malignancies | tumor cells and T-cells | CD161 mAb | Negative | A high-affinity CD161 mAb enhanced key aspects of T-cell function, including cytotoxicity, cytokine production, and proliferation in hematological malignancies. | Alvarez Calderon F, et al.^[11]^ |
| Breast cancer | Tissue | Single-cell RNA-seq | Negative | CD161 compromises T-cell cytotoxicity, inhibiting calcium influx in CTLs, leading to chemoresistance and poor outcomes. | Lao L, et al.^[12]^ |

**Reference:**

1. Huang G, Xiao S, Jiang Z, et al. Machine learning immune-related gene based on KLRB1 model for predicting the prognosis and immune cell infiltration of breast cancer. Front Endocrinol (Lausanne). 2023 Jun 7;14:1185799. doi: 10.3389/fendo.2023.1185799.

2. He JR, Li D, Zhang QX, et al. Inhibiting KLRB1 expression is associated with impairing cancer immunity and leading to cancer progression and poor prognosis in breast invasive carcinoma patients. Aging (Albany NY). 2023 Nov 20;15(22):13265-13286. doi: 10.18632/aging.205239.

3. Hu X, Dong Y, Xie S, et al. Immune checkpoint CD161/LLT1-associated immunological landscape and diagnostic value in oral squamous cell carcinoma. J Pathol Clin Res. 2024 Mar;10(2):e353. doi: 10.1002/cjp2.353.

4. Wei Y, Xu T, Li C, et al. CD161 Characterizes an Inflamed Subset of Cytotoxic T Lymphocytes Associated with Prolonged Survival in Human Papillomavirus-Driven Oropharyngeal Cancer. Cancer Immunol Res. 2023 Mar 1;11(3):306-319. doi: 10.1158/2326-6066.

5. Cha J, Kim DH, Kim G, et al. Single-cell analysis reveals cellular and molecular factors counteracting HPV-positive oropharyngeal cancer immunotherapy outcomes. J Immunother Cancer. 2024 Jun 10;12(6):e008667. doi: 10.1136/jitc-2023-008667.

6.Chen Q, Yin H, Jiang Z, et al. Poor clinical outcomes and immunoevasive contexture in CD161+CD8+ T cells barren human pancreatic cancer. J Immunother Cancer. 2024 Mar 26;12(3):e008694. doi: 10.1136/jitc-2023-008694.

7. Xu S, Xu Y, Chai W, et al. KLRB1 expression is associated with lung adenocarcinoma prognosis and immune infiltration and regulates lung adenocarcinoma cell proliferation and metastasis through the MAPK/ERK pathway. J Thorac Dis. 2024 Jun 30;16(6):3764-3781. doi: 10.21037/jtd-24-8.

8. Chen JL, Wu CY, Luo XY, et al. Down-regulation of KLRB1 is associated with increased cell growth, metastasis, poor prognosis, as well as a dysfunctional immune microenvironment in LUAD. Sci Rep. 2024 May 23;14(1):11782. doi: 10.1038/s41598-024-60414-x.

9.Mathewson ND, Ashenberg O, Tirosh I, et al. Inhibitory CD161 receptor identified in glioma-infiltrating T cells by single-cell analysis. Cell. 2021 Mar 4;184(5):1281-1298.e26. doi: 10.1016/j.cell.2021.01.022.

10. Di W, Fan W, Wu F, et al. Clinical characterization and immunosuppressive regulation of CD161 (KLRB1) in glioma through 916 samples. Cancer Sci. 2022 Feb;113(2):756-769. doi: 10.1111/cas.15236.

11. Alvarez Calderon F, Kang BH, Kyrysyuk O, et al. Targeting of the CD161 inhibitory receptor enhances T-cell-mediated immunity against hematological malignancies. Blood. 2024 Mar 21;143(12):1124-1138. doi: 10.1182/blood.2023022882.

12. Lao L, Zeng W, Huang P, Chen H, Jia Z, Wang P, Huang D, Chen J, Nie Y, Yang L, Wu W, Liu J. CD8+ T cell-Dependent Remodeling of the Tumor Microenvironment Overcomes Chemoresistance. Cancer Immunol Res. 2023 Mar 1;11(3):320-338. doi: 10.1158/2326-6066.CIR-22-0356.
