# Supplementary material for: CD161, a promising prognostic biomarker in hepatocellular carcinoma, correlates with immune infiltration
Source: PeerJ. 2025 Mar 17;13:e19055. doi: 10.7717/peerj.19055 (PMC11925045; doi:10.7717/peerj.19055)
Supplement: Supplemental Information 4 [file peerj-13-19055-s004.docx]

| ALBI | albumin-bilirubin |
| --- | --- |
| CR | complete response |
| DCR | disease control rates |
| GEO | Gene Expression Omnibus |
| GO | Gene Ontology |
| GSVA | Gene set variation analysis |
| HCC | hepatocellular carcinoma |
| IHC | immunohistochemistry |
| IRS | immunoreactivity score |
| KLRB1 | killer cell lectin-like receptor B1 |
| LLT1 | lectin-like transcript 1 |
| MVI | microvascular invasion |
| MHC | major histocompatibility complex |
| NLR | neutrophil-to-lymphocyte ratio |
| OS | overall survival |
| ORR | objective response rates |
| PLR | platelet-to-lymphocyte ratio |
| PVTT | portal vein tumor thrombus |
| PD | progressive disease |
| PR | partial response |
| RFS | relapse free survival |
| SD | stable disease |
| TIME | tumor immune microenvironment |
| TPM | transcripts per kilobase million |
| TCGA | The Cancer Genome Atlas |
| KEGG | Kyoto Encyclopedia of Genes and Genomes |
